# Favorable outcome of high-dose chemotherapy and autologous hematopoietic stem cell transplantation in patients with nonmetastatic osteosarcoma and low-degree necrosis

**DOI:** 10.3389/fonc.2022.978949

**Published:** 2022-09-13

**Authors:** Kyung Taek Hong, Hyun Jin Park, Bo Kyung Kim, Hong Yul An, Jung Yoon Choi, Jung-Eun Cheon, Sung-Hye Park, Han-Soo Kim, Hyoung Jin Kang

**Affiliations:** ^1^ Department of Pediatrics, Seoul National University College of Medicine, Seoul National University Cancer Research Institute, Seoul, South Korea; ^2^ Department of Radiology, Seoul National University College of Medicine, Seoul, South Korea; ^3^ Department of Pathology, Seoul National University College of Medicine, Seoul, South Korea; ^4^ Department of Orthopedic Surgery, Seoul National University College of Medicine, Seoul, South Korea; ^5^ Wide River Institute of Immunology, Hongcheon, South Korea

**Keywords:** osteosarcoma, high-dose chemotherapy, autologous hematopoietic stem cell transplantation, low-degree necrosis, nonmetastatic

## Abstract

**Background:**

A low-degree tumor necrosis after neoadjuvant chemotherapy is a poor prognostic factor for osteosarcoma (OSA). However, the role of high-dose chemotherapy (HDC) and autologous hematopoietic stem cell transplantation in OSA remains controversial. We analyzed the treatment outcomes and prognostic factors of nonmetastatic OSA and compared the HDC and conventional chemotherapy (CC) outcomes of patients with <90% necrosis after neoadjuvant chemotherapy.

**Methods:**

We retrospectively evaluated patients with OSA treated at the Seoul National University Children’s Hospital from 2000 to 2020. Totally, 113 patients with non-metastatic OSA at diagnosis were included. The majority were treated with cisplatin, doxorubicin, and methotrexate as neoadjuvant chemotherapy. This was continued when the postoperative necrosis rate was >90% (good response [GR]), whereas most cases with <90% (poor response [PR]) were changed to chemotherapy. The HDC regimen was composed of melphalan, etoposide, and carboplatin.

**Results:**

The median age at diagnosis was 12.6 years (range, 5.0–20.3), and 61.9% of patients were men. The 5-year event-free survival (EFS) and overall survival (OS) rates were 75.8% and 91.5%, respectively. Among these, 59 and 44 patients were included in the GR and PR groups, respectively. The GR group had a better 5-year EFS rate than the PR group (82.4% vs. 67.3%, p=0.071). Age at diagnosis, sex, tumor site, type of neoadjuvant chemotherapy, and degree of tumor necrosis were not different between the PR-HDC (n=24) and PR-CC (n=20) groups. The 5-year EFS and OS rates in the PR-HDC (n=24) and PR-CC (n=20) groups were 78.6% and 53.6% (p=0.065) and 100% and 76.9% (p=0.024), respectively. In the Cox regression analysis, the PR-CC group (hazard ratio, 4.95; p=0.004) and age ≥12 years (hazard ratio, 2.68; p=0.024) were significant risk factors for 5-year EFS.

**Conclusions:**

HDC showed favorable outcomes in patients with non-metastatic OSA and <90% necrosis after neoadjuvant chemotherapy.

## Introduction

Osteosarcoma (OSA) is a rare tumor that occurs mainly in adolescents and young adults, with 4.4 cases per million at the age of 0–24, accounting for approximately 5% of childhood and adolescent tumors (1, 2). In OSA, surgery without additional chemotherapy has been reported to recur in 90% of patients within 2 years, while systemic chemotherapy is very important in treatment, even if it is a local tumor (3, 4). The 5-year survival rate of OSA rose from approximately 40% to 76% in the 2010s in individuals under the age of 15. 5-year survival rate is 66% at the age of 15–19, which has not increased, particularly since the 1980s (1, 5). In Korea, the survival rate of patients diagnosed in 2007–2011 was 81.5%, although the survival rate was approximately 55.4% in the early 1990s, showing improved treatment outcomes (6).

Prognostic factors for OSA include tumor location, size, metastasis, possibility of surgical resection, and tumor necrosis after chemotherapy (7). It is well known that if the tumor necrosis rate is ≥90% after neoadjuvant chemotherapy, the prognosis is good (8, 9). There have been many studies on which postoperative treatment could improve the prognosis of patients if the tumor necrosis rate after neoadjuvant chemotherapy is <90%. In the EURAMOS-1 trial, patients with OSA who experience poor treatment response to preoperative chemotherapy were divided into a group that received postoperative high-dose methotrexate, cisplatin, and doxorubicin (MAP) and a group that received ifosfamide and etoposide plus MAP. However, the addition of ifosfamide and etoposide was associated with increased toxicity without improving the event-free survival (EFS) (10).

Although many studies have been conducted on high-dose chemotherapy (HDC) and autologous hematopoietic stem cell transplantation (ASCT) in OSA, there is still insufficient evidence to show a clear benefit compared to conventional chemotherapy (CC) (11–13). However, our institution has reported the results of HDC and ASCT using melphalan, etoposide, and carboplatin in high-risk OSA by determining indications based on three risk factors, including tumor necrosis <90% after neoadjuvant chemotherapy, metastasis, progression on therapy, or relapse, which showed promising outcomes (9, 14). Here, we retrospectively analyzed the treatment outcome of patients with local OSA without metastasis at the time of diagnosis and investigated the feasibility and safety of HDC and ASCT in patients with necrosis <90% after neoadjuvant chemotherapy.

## Methods

### Patients

**Figure 1:**
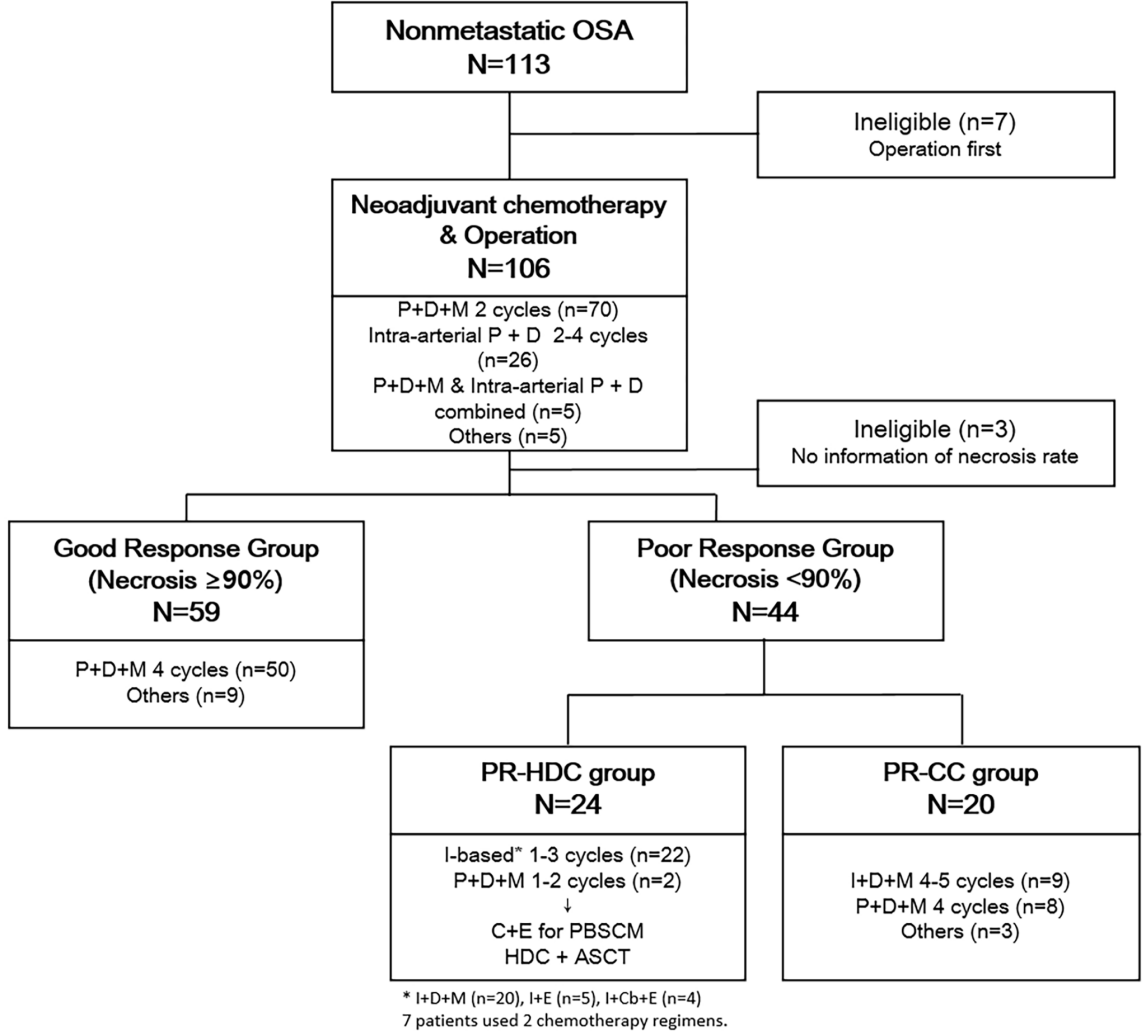
Overall treatment scheme and classification of patients. ASCT, autologous hematopoietic stem cell transplantation; C, cyclophosphamide; Cb, carboplatin; D, doxorubicin; E, etoposide; I, ifosfamide; P, cisplatin; PR-CC, poor response-chemotherapy only; PR-HDC, poor response-high dose chemotherapy.

We retrospectively reviewed the data of 113 patients diagnosed with non-metastatic OSA treated at Seoul National University Children’s Hospital from 2000 to 2020. Among them, seven patients who had undergone surgery and three patients who did not have information on the necrosis rate after neoadjuvant chemotherapy were excluded, and a total of 103 patients were included for further analysis (**Figure 1**). All the patients underwent a bone scan and chest computed tomography at the initial diagnosis, and no metastasis was found. The patient completed the planned neoadjuvant chemotherapy and underwent surgery, and there were no events for at least one month after the operation. Patients with a necrosis rate of ≥90% and <90% after neoadjuvant chemotherapy were defined as the good response (GR) and poor response (PR) groups. The overall patient classification and treatment scheme are shown in **Figure 1**. The Institutional Review Board approved the procedure for reviewing medical records, and obtaining consent was waived (H-1911-169-1082).

### Chemotherapy and treatment plan

Most patients (n=70) received intravenous cisplatin, doxorubicin, and high-dose methotrexate [Children’s Cancer Group-7921, regimen A (15)] as neoadjuvant chemotherapy, 26 received intra-arterial cisplatin plus intravenous doxorubicin, and 5 received combined intravenous and intra-arterial chemotherapy. Of the 103 patients who were able to confirm the postoperative necrosis rate, most of the GR group (n=50) were continuously administered regimen A. Among the PR group (n=44), the majority changed to ifosfamide-based chemotherapy [based on Children’s Cancer Group-7921, regimen B (15)]. From November 2007, HDC and ASCT were performed after peripheral blood stem cell (PBSC) mobilization if patients and guardians agreed. Otherwise, adjuvant CC was continued. An overview of the chemotherapy regimens is presented in **Figure 1**.

Autologous PBSCs were mobilized either with chemotherapy (cyclophosphamide plus etoposide) or plerixafor using granulocyte colony-stimulating factor (16). HDC consisted of melphalan 140 mg/m^2^ on day − 7 and 70 mg/m^2^ on day − 6, etoposide 200 mg/m^2^ and carboplatin 400 mg/m^2^ from days − 8 to − 5. Post-ASCT management was conducted according to institutional guidelines (14, 17).

### Definition

Neutrophil engraftment and platelet engraftment days were calculated as the first three days with neutrophil count >0.5×10^9^/L and platelet count >20×10^9^/L, respectively, without transfusion for at least 7 days. The toxicity was graded according to the National Cancer Institute Common Toxicity Criteria (v4.03).

### Statistical analysis

Categorical variables were compared using the chi-square test, and continuous variables were compared using Student’s t-test or one-way analysis of variance. Events were defined as deaths or relapses. For survival analysis, overall survival (OS) was defined as the time from diagnosis to death from any cause, and EFS was defined as the time from diagnosis to first relapse or death. Patients who did not experience an event were censored at the last follow-up visit. Survival was analyzed using the Kaplan–Meier method. Differences in the survival rates were determined using the log-rank test. A Cox proportional hazard regression model was used for multivariate analysis of prognostic factors affecting survival; independent variables (p<0.2) were included in this model. Statistical significance was set at P < 0.05. Statistical analyses were conducted using the R version 3.2.2 (www.r-project.org) and SPSS 23.0 (IBM-SPSS, Armonk, NY, USA) softwares.

## Results

### Patient characteristics

The clinical characteristics of the patients are summarized in **Table 1**. All patients were diagnosed with non-metastatic OSA, with a median age of 12.6 years (range, 5.0–20.3) and 61.9% were men. The median follow-up period was 7.7 years (range, 0.3–21.3 years). The most common tumor location was the distal femur (46.9%), followed by the proximal tibia (18.6%) and the humerus (12.4%). Thirty-one patients (27.4%) were treated at least once with neoadjuvant intra-arterial chemotherapy, while four patients had *RB1* germline mutations and had previously been treated for retinoblastoma (**Table 1**).

**Table 1:** Patient characteristics.

Characteristics	n=113
Age at diagnosis, median (range)	12.6 (5.0-20.3)
Sex	
male	70 (61.9%)
female	43 (38.1%)
Primary site	
distal femur	53 (46.9%)
proximal femur	4 (3.5%)
proximal tibia	21 (18.6%)
distal tibia	6 (5.3%)
humerus	14 (12.4%)
others^a^	15 (13.3%)
Histology	
osteoblastic	68 (60.2%)
chondroblastic	14 (12.4%)
others^b^	4 (3.9%)
unknown	27 (23.9%)
Intraarteral chemotherapy as a neoadjuvant chemotherapy, yes	31 (27.4%)
previous retinoblastoma history, yes	4 (3.5%)
Necrosis rates after neoadjuvant chemotherapy	
≥90%	59 (52.2%)
50-89%	19 (16.8%)
10-49%	19 (16.8%)
<10%	6 (5.3%)
not applicable	10 (8.8%)
Follow-up year, median (range)	7.7 (0.3-21.3)

a 2 distal fibula, 2 rib, 2, mandible, 2 ileum, 1 proximal fibula, 1 vertebral body (T9), 1 radius, 1 sacrum, 1 temporal bone, 1 occipital bone, 1 palate.

b 1 fibroblastic, 1 periosteal, 1 telangiectatic, 1 giant cell rich.

Among them, seven patients underwent surgery first, and three had no information on necrosis rate. Therefore, the remaining 103 patients were included in further analysis based on the necrosis rate after neoadjuvant chemotherapy. Fifty-nine patients had a tumor necrosis rate of >90% after neoadjuvant chemotherapy, and they were classified into the GR group, while 44 patients were classified into the PR group. The median age at diagnosis, sex, tumor location, histology, and median follow-up time did not differ between the GR and PR groups. All patients, except for one, underwent wide excision or limb salvage surgery, and all margins were negative. One remaining patient had a tumor on T9, and only margin-positive tumor excision was performed. A comparison of the characteristics of the two groups is presented in **Supplementary Table 1**.

### Outcome

**Figure 2:**
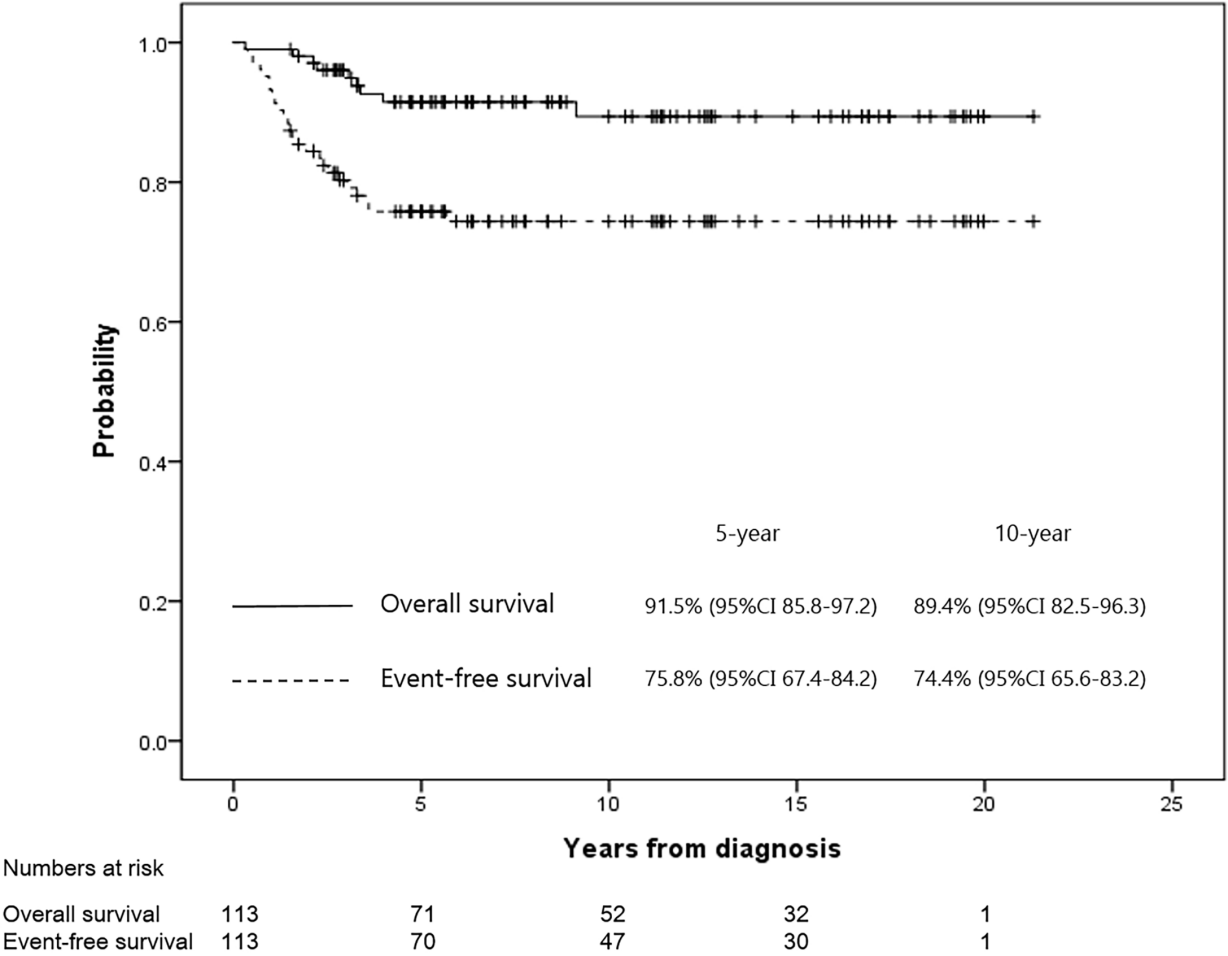
The EFS and OS rates for all patients were 75.8% and 91.5% at 5 years, and 74.4% and 89.4% at 10 years, respectively.

The 5- and 10-year OS rates in all patients were 91.5% (95% confidence interval [CI], 85.8–97.2) and 89.4% (95% CI, 82.5–96.3), respectively. Moreover, the 5- and 10-year EFS rates in all patients were 75.8% (95% CI, 67.4–84.2) and 74.4% (95% CI, 65.6–83.2), respectively (**Figure 2**). Only one case of a late event occurred five years after diagnosis in all patients. When all patients were divided into the GR (n=59) and PR (n=44) groups according to the necrosis rate, the 5-year EFS rates of the GR and PR groups were 82.4% (95% CI, 72.4–92.4) versus 67.3% (95% CI, 53.2–81.4; P=0.071), respectively, and the 5-year OS rates were 92.9% (95% CI, 86.2–99.6) versus 89.3% (95% CI, 79.6–99.6; P=0.766), respectively. Among the four patients with a history of retinoblastoma (2 GR and 2 PR groups, respectively), two patients were event-free at the data cut-off date (follow-up time 5.3 and 5.4 years, respectively), and one patient died due to septic shock during chemotherapy. The remaining patient relapsed after 3 years and received a second surgery and salvage chemotherapy.

### Sub-analysis of the PR group

In the PR group (n=44), 24 patients underwent HDC and ASCT (PR-HDC), and 20 patients received only adjuvant CC (PR-CC). A comparison of the groups is presented in **Table 2**. The median age at diagnosis, tumor location, histology, necrosis rate, and median follow-up time did not differ between the groups; however, more men were included in the PR-HDC group than women (P=0.019).

**Table 2:** Comparison of characteristics of the poor response groups.

Characteristics	PR-HDC (n=24)	PR-CC (n=20)	p value
Age at diagnosis, median (range)	12.7 (5.3-16.8)	11.6 (6.3-16.1)	0.268
Sex			0.019
male	19 (79.2%)	9 (45.0%)	
female	5 (20.8%)	11 (55.0%)	
Primary site			0.935
distal femur	10 (41.7%)	9 (45.0%)	
proximal femur	1 (4.2%)	1 (5.0%)	
proximal tibia	5 (20.8%)	4 (20.0%)	
distal tibia	1 (4.2%)	1 (5.0%)	
humerus	5 (20.8%)	2 (10.0%)	
others	2 (8.3%)	3 (15.0%)	
histology			0.437
osteoblastic	20 (83.3%)	13 (65.0%)	
chondroblastic	2 (8.3%)	4 (20.0%)	
others	1 (4.2%)	2 (10.0%)	
unknown	1 (4.2%)	1 (5.0%)	
Intraarteral chemotherapy as a neoadjuvant chemotherapy, yes	2 (8.3%)	6 (30.0%)	0.064
previous retinoblastoma history, yes	1 (4.2%)	1 (5.0%)	0.895
Necrosis rates after neoadjuvant chemotherapy			0.348
≥90%	0 (0.0%)	0 (0.0%)	
50-89%	8 (33.3%)	11 (55.0%)	
10-49%	12 (50.0%)	7 (35.0%)	
<10%	4 (16.7%)	2 (10.0%)	
follow-up year, median (range)	6.9 (1.5-13.9)	5.0 (2.1-21.3)	0.261

PR-CC, Poor response-chemotherapy only group; PR-HDC, Poor response-high dose chemotherapy group.

**Figure 3:**
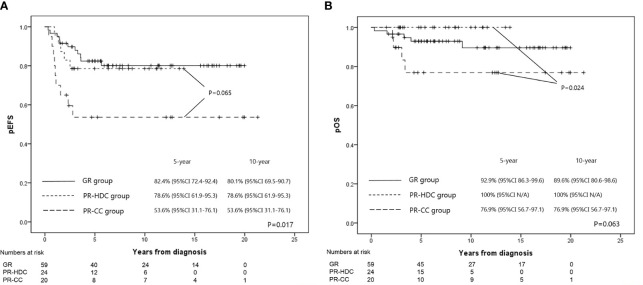
The EFS rates **(A)** and OS rates **(B)** at 5 years for GR, PR-HDC, and PR-CC groups were 82.4% versus 78.6% versus 53.6% (P=0.017), and 89.6% versus 100% versus 76.9% (P=0.063), respectively.

The 5-year EFS and OS rates of the PR-HDC and PR-CC groups were 78.6% (95% CI, 61.9–95.3) versus 53.6% (95% CI, 31.1–76.1; P=0.065) and 100% versus 76.9% (95% CI, 56.7–97.1; P=0.024), respectively (**Figure 3A, B**). In the PR group, there was no non-relapse mortality and all events were recurrences. Nine patients (45.0%) in the PR-CC group and five patients (20.8%) in the PR-HDC group had recurrence. The relapse sites and characteristics, an overview of salvage treatment, and outcomes are summarized in **Table 3**. Notably, most of the cases of recurrence in the PR-HDC group were solitary lesions, in contrast to the PR-CC group, and all relapsed patients in the PR-HDC group were successfully treated and survived without recurrence.

**Table 3:** Relapse sites and salvage treatments of the poor response groups.

Group	No.	Relapse site & characteristics	Salvage treatment	Outcome (Months from relapse)
PR-CC	1	Lung, solitary nodule	Op & chemotherapy (unknown)	T-MDS, Follow-up loss (33)
	2	Lung & pleura, multiple	chemotherapy (IE, ICb)	DOD (7)
	3	Lung, multiple	Op & chemotherapy (BCA, IE, ICb, CbCE 4, CTE)	NED (223)
			HDC, ASCT	
	4	Lung, multiple	Op & chemotherapy -> IE, ICb, DCb, CbCE, CT, CTE, GDo)	DOD (16)
	5	Lung, solitary nodule	Op & chemotherapy (ICbE, GDo, BCD)	NED (138)
	6	Lung & bone, multiple	chemotherapy (DP, BCA, CTE, GD, ICbE)	follow-up loss with disease (20)
	7	Lung, multiple -> Skull & brain	Op & RT + chemotherapy (ICbE)	follow-up loss with disease (32)
	8	Lung, multiple	Op & RT + chemotherapy (GDo) +RT	DOD (14)
	9	primary site(ankle)	Op	NED (2)
PR-HDC	1	Lung, solitary	Op & chemotherapy (ICbE)	NED (92)
	2	Lung, solitary	Op & chemotherapy (GDo, CE)	NED (52)
	3	Op site, tiny mass	Op & chemotherapy (ICbE)	NED (27)
	4	Lung, solitary	Op & chemotherapy (ICbE, GDo)	NED (25)
	5	Lung, solitary	Op	NED (19)

A, actinomycin D; ASCT, autologous hematopoietic stem cell transplantation; B, bleomycin; C, cyclophosphamide; Cb, carboplatin; D, doxorubicin; Do, docetaxel; DOD, dead of disease; E, etoposide; G, gemcitabine; I, ifosfamide; NED, no evidence of disease; Op, operation; P, cisplatin; PR-CC, poor responsechemotherapy only; PR-HDC, poor response-high-dose chemotherapy; T, topotecan; T-MDS, therapy-related myelodysplastic syndrome.

All patients in the PR-HDC group achieved successful neutrophil and platelet engraftment. The median times of neutrophil and platelet engraftment were 9 (range, 8–11) and 15 (range, 11–40) days, respectively. The median infused post-thawing mononuclear and CD34+ cell counts were 8.68 × 10^8^/kg and 4.04 × 10^6^/kg, respectively. Complications associated with ASCT, such as hepatic veno-occlusive disease, thrombotic microangiopathy, or transfer to intensive care units, did not occur. More detailed information on the HDC-PR group is provided in **Table 4**. With regard to long-term complications, 3 premature ovarian failures, 2 hearing impaired (1 grade 1, 1 grade 2), 2 hypothyroidisms (grade 2), and 2 proteinurias (1 grade 1, 1 grade 2) occurred in the HDC group, and 2 Therapy-related myelodysplastic syndromes, 1 chronic kidney disease (grade 4), 2 proteinurias (grade 2), and 1 diabetes mellitus in the CC group.

**Table 4:** Stem cell dose, engraftment and adverse events in the PR-HDC group.

Characteristics		N=24
Infused post-thawing cell dose		
Mononuclear cells		8.68 × 10^8^ per recipient body weight (kg)
CD34^+^ cells		4.04 × 10^6^ per recipient body weight (kg)
Engraftment day		
Neutrophil, median (range)		9 (8-11)
Platelet, median (range)		15 (11-40)
Adverse events related to the ASCT		
Febrile neutropenia		23 (95.8%)
Serum aminotransferases elevation		
	Grade 1	6 (25.0%)
	Grade 2	2 (8.3%)
	Grade 3	15 (62.5%)
	Grade 4	1 (4.2%)
Total bilirubin elevation		
	Grade 1	2 (8.3%)
	Grade 2	4 (16.7%)
Serum Creatinine elevation		
	Grade 1	4 (16.7%)
	Grade 2	4 (16.7%)
CMV reactivation		3 (12.5%)
CMV disease		0 (0.0%)
Hepatic veno-occlusive disease		0 (0.0%)
Thrombotic microangiopathy		0 (0.0%)
Transfer to intensive care unit		0 (0.0%)

ASCT, Autologous hematopoietic stem cell transplantation; CMV, cytomegalovirus; PR-HDC, poor response-high-dose chemotherapy.

### Prognostic factors

Among the variables, including necrosis rate (≥90%, 50–89%, and <50%), tumor sites, sex, and intra-arterial chemotherapy, there was no statistically significant difference in the univariate analyses of EFS and OS. However, the PR-CC group was associated with poor EFS (P=0.017). In the multivariate analysis, PR-CC was a significant poor prognostic factor for EFS (P=0.004) and OS (P=0.008), and age at diagnosis of >12 years was a significant poor prognostic factor for EFS (P=0.024) (**Table 5**).

**Table 5:** Univariate and multivariate analyses of event-free and overall survivals.

			Event-free survival						Overall survival					
		n	Event	5-year rate	P Value	Exp(B)	95% CI	p value	Event	5-year rate		Exp(B)	95$ CI	p value
Necrosis rate					0.197						0.624			
	≥90%	59	11	82.4 ± 5.1					5	92.9 ± 3.4				
	50-89	19	6	66.5 ± 11.3					1	93.8 ± 6.1				
	<50	25	8	67.5 ± 9.5					3	85.9 ± 7.8				
Age at diagnosis					0.082			0.024			0.230			0.073
	≤12	51	9	84.0 ± 5.2		1			3	93.8 ± 3.5		1		
	>12	52	16	67.8 ± 6.7		2.680	1.138-6.311		6	88.9 ± 4.7		4.166	0.873-19.876	
Primary site					0.483						0.408			
	femur	57	11	80.0 ± 5.4					3	94.6 ± 3.0				
	tibia	27	8	73.9 ± 8.5					3	92.3 ± 5.2				
	humerus	10	4	57.1 ± 16.4					2	91.4 ± 17.1				
	others	9	2	74.1 ± 16.1					1	83.3 ± 15.2				
Sex					0.881						0.751			
	male	64	16	75.2 ± 5.6					6	91.2 ± 3.8				
	female	39	9	76.4 ± 6.9					3	91.9 ± 4.5				
Treatment group					0.017			0.110			0.063			0.031
	GR	59	11	82.4 ± 5.1		1			5	92.9 ± 3.4		1		
	PR-HDC	24	5	78.6 ± 8.5		1.353	0.449-4.077	0.591	0	100		0		0.972
	PR-CC	20	9	53.6 ± 11.5		4.950	1.665-14.715	0.004	4	76.9 ± 10.3		9.080	1.766-16.689	0.008
intra-arterial chemotherapy as neoadjuvant					0.580						0.373			
	yes	31	9	74.2 ± 7.9					2	93.1 ± 4.8				
	no	72	16	76.3 ± 5.2					7	88.6 ± 4.1				

CI, confidence interval; GR, good response; PR-CC, poor response-chemotherapy only; PR-HDC, poor response-high dose chemotherapy.

## Discussion

Our study shows promising treatment outcomes for HDC and ASCT using melphalan, etoposide, and carboplatin in patients with non-metastatic osteosarcoma when the tumor necrosis rate is <90% after neoadjuvant chemotherapy. In this study, the PR group showed a tendency toward lower EFS than the GR group; however, the PR-HDC group showed 100% OS, which translated to improved overall treatment outcomes in the PR group. In particular, the PR-HDC group showed EFS and OS similar to those of the GR group, and the recurrences that occurred in the PR-HDC group were more successfully salvaged than those in the PR-CC group.

Various efforts have been made to improve the treatment outcomes of patients with localized OSA who show lower degrees of necrosis after preoperative chemotherapy. In a previous study, patients with lower degrees of necrosis were administered additional cisplatin after surgery; however, there was no significant difference in the outcome (18). The EURAMOS trial has shown that adding ifosfamide and etoposide does not improve prognosis (10). In line with this study, a report by the Children’s Oncology Group that added higher cumulative doses of doxorubicin or ifosfamide plus etoposide for patients with lower degrees of necrosis did not show improvement in outcomes (19). In addition, studies applying HDC using melphalan alone or carboplatin plus etoposide did not show an improved outcome (11, 13).

Our institution demonstrated the feasibility of using HDC in patients with OSA in a previous pilot study (14). We followed the same strategy for patients with lower degrees of necrosis after neoadjuvant chemotherapy. Eight patients (33.3%) failed PBSC mobilization after chemotherapy while PBSC were able to be collected successfully following treatment with plerixafor. All patients were safely engrafted after ASCT and recovered without serious ASCT-related adverse events. We used a melphalan/etoposide/carboplatin regimen, which was different from the studies that conducted HDC in OSA. This regimen has already been used for a variety of childhood cancers and is familiar to pediatricians (20–22). All of these medications are known to be effective in OSA, and we have confirmed through a previously reported pilot study that the melphalan/etoposide/carboplatin regimen showed good treatment results, especially in localized OSA patients with low-degree necrosis (14). Although selection bias should be considered, in which only patients with transplantable conditions can be included in the PR-HDC group, our study showed that HDC is feasible as a concept of consolidation treatment in OSA.

It is noteworthy that the relapsed patients in the PR-HDC group had only a solitary lesion, and thus, all of them survived with metastasectomy and salvage chemotherapy. Although EFS still needs to be improved, HDC and ASCT could improve the OS of localized OSA with lower degrees of necrosis.

However, this study was a retrospective analysis for 20 years, which is limited in that it included patients who received heterogeneous chemotherapies. In particular, 27.4% of the patients who received intra-arterial neoadjuvant chemotherapy were included. In this study, more patients who received intra-arterial chemotherapy were included in the PR-CC group, although there was no difference in EFS and OS compared with intravenous chemotherapy, as in previous studies (23). In addition, this study included only nonmetastatic OSA, and it was difficult to evaluate the role of HDC and ASCT in cases of recurrence or metastasis at initial diagnosis. In particular, attention should be paid to the interpretation of our study in consideration of previous studies that did not show improved outcomes even if HDC was used (11, 13). However, this study is different from the previous studies in that we used the melphalan/etoposide/carboplatin regimen and analyzed only nonmetastatic OSA patients who showed low-degrees necrosis after neoadjuvant chemotherapy.

In conclusion, this study reported 10-year EFS and OS rates of 74.4% and 89.4%, respectively, in 113 patients with non-metastatic OSA who were treated for >20 years in a single institution. Moreover, HDC with melphalan, etoposide, and carboplatin showed favorable outcomes in patients with non-metastatic osteosarcoma, with necrosis of <90% after neoadjuvant chemotherapy. Further prospective studies are required to confirm the role of HDC and ASCT in OSA.

## Data availability statement

The raw data supporting the conclusions of this article will be made available by the authors, without undue reservation.

## Ethics statement

The studies involving human participants were reviewed and approved by Seoul National University Hospital. Written informed consent for participation was not provided by the participants’ legal guardians/next of kin because: the Institutional Review Board approved the procedure for reviewing medical records, and obtaining consent was waived.

## Author contributions

KTH performed the analysis and wrote and reviewed the article. HJP, BKK, HYA, and JYC collected the data and reviewed the article. J-EC, S-HP, and H-SK reviewed the article. HJK supervised, wrote, and reviewed the article. All authors contributed to the article and approved the submitted version.

## Funding

This research was supported by a grant (20182MFDS443) from Ministry of Food and Drug Safety in 2020 and supported by grant no 0320200110 from the SNUH Research Fund.

## Conflict of interest

The authors declare that the research was conducted in the absence of any commercial or financial relationships that could be construed as a potential conflict of interest.

## Publisher’s note

All claims expressed in this article are solely those of the authors and do not necessarily represent those of their affiliated organizations, or those of the publisher, the editors and the reviewers. Any product that may be evaluated in this article, or claim that may be made by its manufacturer, is not guaranteed or endorsed by the publisher.
