# Supplementary material for: Favorable outcome of high-dose chemotherapy and autologous hematopoietic stem cell transplantation in patients with nonmetastatic osteosarcoma and low-degree necrosis
Source: Front Oncol. 2022 Sep 13;12:978949. doi: 10.3389/fonc.2022.978949 (PMC9513349; doi:10.3389/fonc.2022.978949)
Supplement: Supplementary file 1 [file Table_1.docx]

**Table S1. Comparison of characteristics of the good response and the poor response groups**

|  |  | Good Response Group (n=59) | Poor Response Group (n=44) | p value |
| --- | --- | --- | --- | --- |
| Age at diagnosis | | 12.3 (5.0-17.3) | 12.1 (5.3-16.8) | 0.954 |
| Sex | |  |  | 0.786 |
|  | male | 36 (61.0%) | 28 (63.6%) |  |
|  | female | 23 (39.0%) | 16 (36.4%) |  |
| Site | |  |  | 0.419 |
|  | distal femur | 34 (57.6%) | 19 (43.2%) |  |
|  | proximal femur | 2 (3.4%) | 2 (4.5%) |  |
|  | priximal tibia | 12 (20.3%) | 9 (20.5%) |  |
|  | distal tibia | 4 (6.8%) | 2 (4.5%) |  |
|  | humerus | 3 (5.1%) | 7 (15.9%) |  |
|  | others | 4 (6.8%) | 5 (11.4%) |  |
| Histology | |  |  | 0.01 |
|  | Osteoblastic | 32 (54.2%) | 33 (75.0%) |  |
|  | Chondroblastic | 6 (10.2%) | 6 (13.6%) |  |
|  | Others | 1 (1.7%) | 3 (6.8%) |  |
|  | Unknown | 20 (33.9%) | 2 (4.5%) |  |
| Intraarteral chemotherapy as a neoadjuvant chemotherapy, yes | | 23 (39.0%) | 8 (18.2%) | 0.023 |
| Previous retinoblastoma history, yes | | 2 (3.4%) | 2 (4.5%) | 0.764 |
| Necrosis | |  |  | <0.001 |
|  | ≥90% | 59 (100%) | 0 (0.0%) |  |
|  | 50-89% | 0 (0.0%) | 19 (43.2%) |  |
|  | 10-49% | 0 (0.0%) | 19 (43.2%) |  |
|  | <10% | 0 (0.0%) | 6 (13.6%) |  |
| Follow-up year, median (range) | | 8.7 (0.3-20.0) | 6.3 (1.5-21.3) | 0.084 |
